# Neutralizing Antibodies Inhibit HIV-1 Infection of Plasmacytoid Dendritic Cells by an FcγRIIa Independent Mechanism and Do Not Diminish Cytokines Production

**DOI:** 10.1038/srep05845

**Published:** 2014-08-18

**Authors:** Alexandre Lederle, Bin Su, Vincent Holl, Julien Penichon, Sylvie Schmidt, Thomas Decoville, Géraldine Laumond, Christiane Moog

**Affiliations:** 1 INSERM U1109, Fédération de Médecine Translationnelle de Strasbourg (FMTS), Université de Strasbourg, 3 rue Koeberlé, 67000 Strasbourg, France; 2 Current address: INSERM U1110, FMTS, Université de Strasbourg, 3 rue Koeberlé, 67000 Strasbourg, France; 3 Deceased

## Abstract

Plasmacytoid dendritic cells (pDC) expressing FcγRIIa are antigen-presenting cells able to link innate and adaptive immunity and producing various cytokines and chemokines. Although highly restricted, they are able to replicate HIV-1. We determined the activity of anti-HIV-1 neutralizing antibodies (NAb) and non-neutralizing inhibitory antibodies (NNIAb) on the infection of primary pDC by HIV-1 primary isolates and analyzed cytokines and chemokines production. Neutralization assay was performed with primary pDC in the presence of serial antibodies (Ab) concentrations. In parallel, we measured the release of cytokines and chemokines by ELISA and CBA Flex assay. We found that NAb, but not NNIAb, inhibit HIV-1 replication in pDC. This inhibitory activity was lower than that detected for myeloid dendritic cells (mDC) infection and independent of FcγRIIa expressed on pDC. Despite the complete protection, IFN-α production was detected in the supernatant of pDC treated with NAb VRC01, 4E10, PGT121, 10-1074, 10E8, or polyclonal IgG44 but not with NAb b12. Production of MIP-1α, MIP-1β, IL-6, and TNF-α by pDC was also maintained in the presence of 4E10, b12 and VRC01. These findings suggest that pDC can be protected from HIV-1 infection by both NAb and IFN-α release triggered by the innate immune response during infection.

Vaccination strategy aims to induce human immunodeficiency virus (HIV) specific antibodies (Ab) that inhibit the infection of HIV target cells at the very beginning of viral transmission^1^,^2^,^3^. Humoral response against HIV has been well studied and allowed to characterize neutralizing antibodies (NAb) described for their ability to efficiently neutralize a broad range of HIV-1 strains *in vitro*. Recently, novel broadly NAb (e.g.VRC01, PGT family, 3BNC117, 10-1074, etc) capable of neutralizing a large spectrum of HIV-1 isolates of various clades have been discovered, in addition to the previously well characterized NAb b12, 2G12, 447-52D, 2F5, 4E10^4^,^5^,^6^,^7^,^8^,^9^,^10^,^11^. These Ab efficiently inhibit HIV-1 primary isolates or pseudoviruses *in vitro* in conventional neutralization assay with PBMC or TZM-bl cells. In addition to neutralization, numerous *in vivo* studies suggest that, other mechanisms may help to protect against HIV acquisition^12^,^13^,^14^,^15^,^16^. The passive transfer of the IgG1 b12 mutant LALA (unable to bind Fcγ receptor [FcγR]) was significantly less efficient in protecting macaques from a SHIV vaginal challenge than the wild-type IgG1 b12, showing that Fcγ portion of NAb participates in the protection of animals against infection^12^,^13^. Our group has reported an additional mechanism of inhibition dependent on the FcγRs expressed on antigen-presenting cells (APC)^15^,^17^,^18^,^19^,^20^,^21^. Various HIV-1 target cells, including monocyte-derived dendritic cells (MoDC), Langerhans cells, interstitial dendritic cells (DC) and macrophages were efficiently protected from HIV-1 infection by NAb. Furthermore, additional anti-HIV-1 Ab, which do not exhibit classical neutralizing activity, inhibit the HIV-1 infection of these APC by the FcγR-dependent mechanism only^18^,^20^,^21^. Such Ab are referred to as non-neutralizing inhibitory antibodies (NNIAb).

Despite the expression of high level of restriction factors^22^, plasmacytoid dendritic cells (pDC) can be infected by R5 and X4-tropic HIV-1 isolates^23^,^24^. Unlike myeloid dendritic cells (mDC), human pDC only express FcγRIIa and not the other FcγRs^25^. Moreover, pDC produce cytokines and chemokines, and in particular large amounts of type I interferon (IFN) in the presence of HIV^26^. These cells play an important role in innate immune response (reviewed in^27^,^28^) and in the triggering of the adaptive immune response by presenting antigens, although with less efficiency than mDC (reviewed in^29^,^30^).

In this study, we assessed the capacity of Ab (NAb and NNIAb) to inhibit HIV-1 replication in pDC, and analyzed the involvement of FcγRIIa in this inhibition. In addition, the production of IFN-α, MIP-1α, MIP-1β, IL-6, and TNF-α by pDC was determined following inhibition of pDC infection by HIV-1 specific Ab.

## Results

### Purified Primary pDC Express FcγRII and are Infected by HIV-1

**Figure 1:**
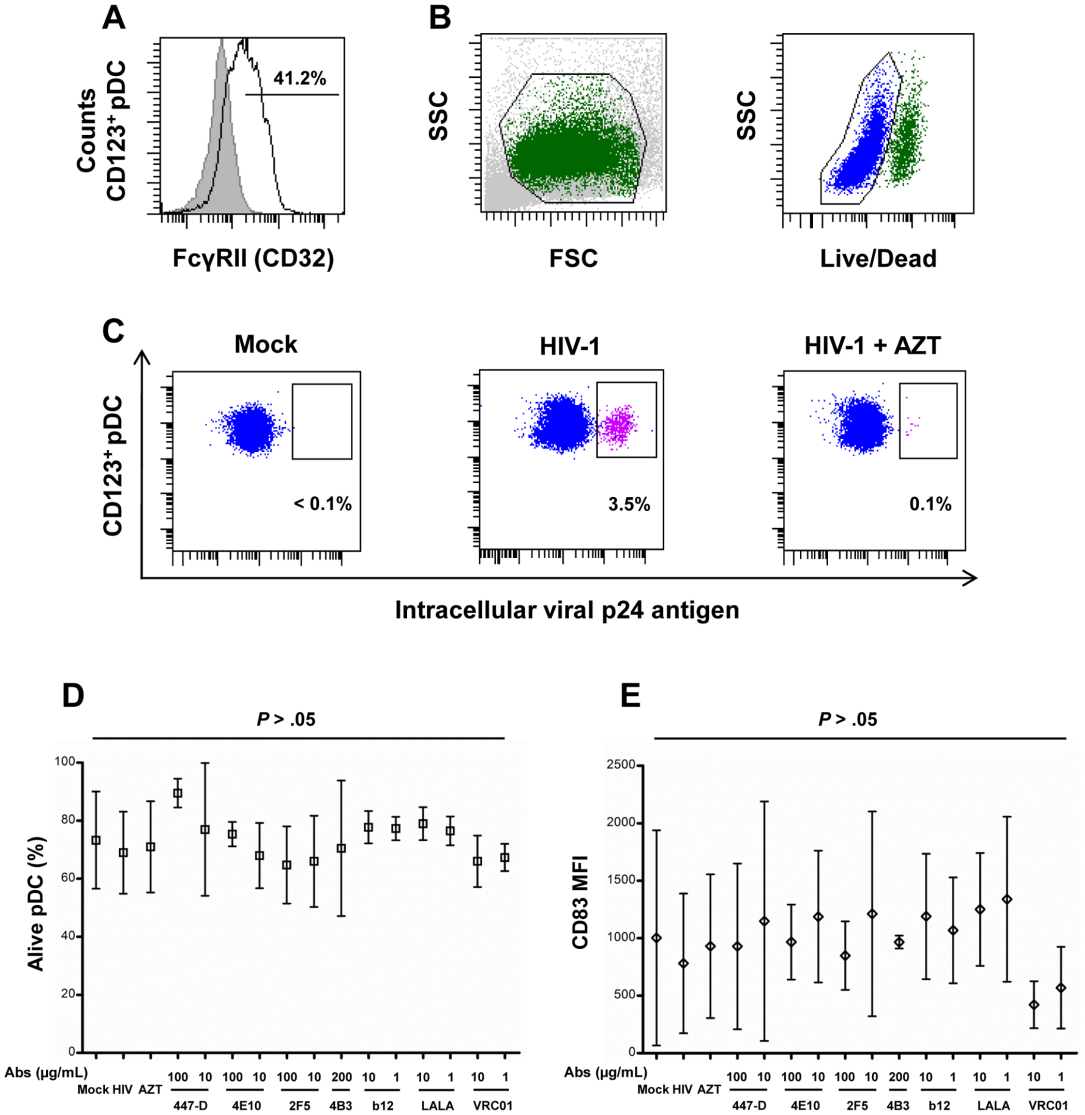
Primary pDC express FcγRII and are productively infected by HIV-1. (A) The percentage of FcγRII-positive cells was determined in living CD123-positive pDC; isotype controls are in grey and FcγRII staining is the black line. (B) Dot plot representation of pDC analysis by flow cytometry. Events were sorted using forward and side scatter (B, left), doublet events were excluded and only living cells, selected by Live/Dead exclusion, were included in the analysis (B, right). (C) Productive infection was detected by intracellular immunolabeling of the p24 HIV antigen in living CD123-positive pDC (in blue). Dot plots show non-infected controls (Mock, left), HIV-1_BaL_ replication (middle, in pink), and negative controls for HIV-1_BaL_ replication with reverse transcriptase inhibitor AZT (5 µM) (right). (D) Viability of primary pDC during neutralization assay: primary pDC were stained with Live/Dead to ensure viability of the cells. Means of the percentage of alive CD123-positive cells is represented for non-infected control (Mock), HIV-1_BaL_ infected cells (HIV), infected cells treated with the reverse transcriptase inhibitor AZT (5 µM), and cells were infected in the presence of various concentrations of anti-HIV Abs. (E) Maturation of primary pDC during neutralization assay: maturation of primary pDC was analyzed by CD83 labeling. Mean of the CD83 MFI (mean of fluorescence intensity) measured in each experiment is represented for pDC treated under the same conditions described in (D). One-way ANOVA showed no significant difference between these conditions. Data are means ± standard deviation (SD) of independent experiments performed with primary pDC from at least three different healthy donors. Groups were compared by one-way ANOVA (Kruskal-Wallis test), with *P* < .05 considered significant.

Before analyzing the inhibitory activity of Ab and the role of FcγRII in this inhibition, the expression of FcγRII by living CD123-positive pDC and the infection of these cells were firstly evaluated by flow cytometry. We found that 41.2% of living CD123-positive pDC expressed FcγRII after BDCA-4 positive selection (Fig. 1A). This result agrees with previous reports showing that 30 to 50% of FcγRIIa, and not other FcγRs, was detected on pDC present in the population isolated from blood circulation^25^. Thus, BDCA-4 positive selection was further used in this study to investigate the role of FcγRIIa in the inhibition of HIV-1 replication by Ab. HIV-1 infection of pDC were detected by flow cytometry according to the size and the structure (Fig. 1B, left dot plot); the living population (Fig. 1B, right dot plot) was selected and the percentage of p24^+^ cells among the CD123^+^ population was determined (Fig. 1C, left dot plot, mock condition). 3.5% of pDC were positive for p24 after 48 h of culture with HIV-1_BaL_ primary isolate, compared to only 0.1% of the AZT controls (Fig. 1C). Inhibition of HIV-1_BaL_ infection of the pDC was analyzed for a panel of anti-HIV-1 Ab by flow cytometry as described above. The viability of pDC was similar for the different healthy blood donors and for all experimental conditions, i.e.: approximately 70% of pDC were alive after 48 h of incubation with or without HIV-1 and with or without anti-HIV-1 specific Ab (Fig. 1D). Expression of the maturation marker CD83 at the surface of pDC varied depending on the donors but did not differ significantly after infection and in the presence of anti-HIV-1 Ab (Fig. 1E). Therefore, neither HIV-1 nor Ab influenced the viability and the maturation of the pDC. We further used these experimental conditions to study the inhibitory activity of anti-HIV-1 specific Ab and the role of FcγRIIa in this inhibition.

### Neutralizing Antibodies Inhibit HIV-1 Infection of Primary pDC

**Figure 2:**
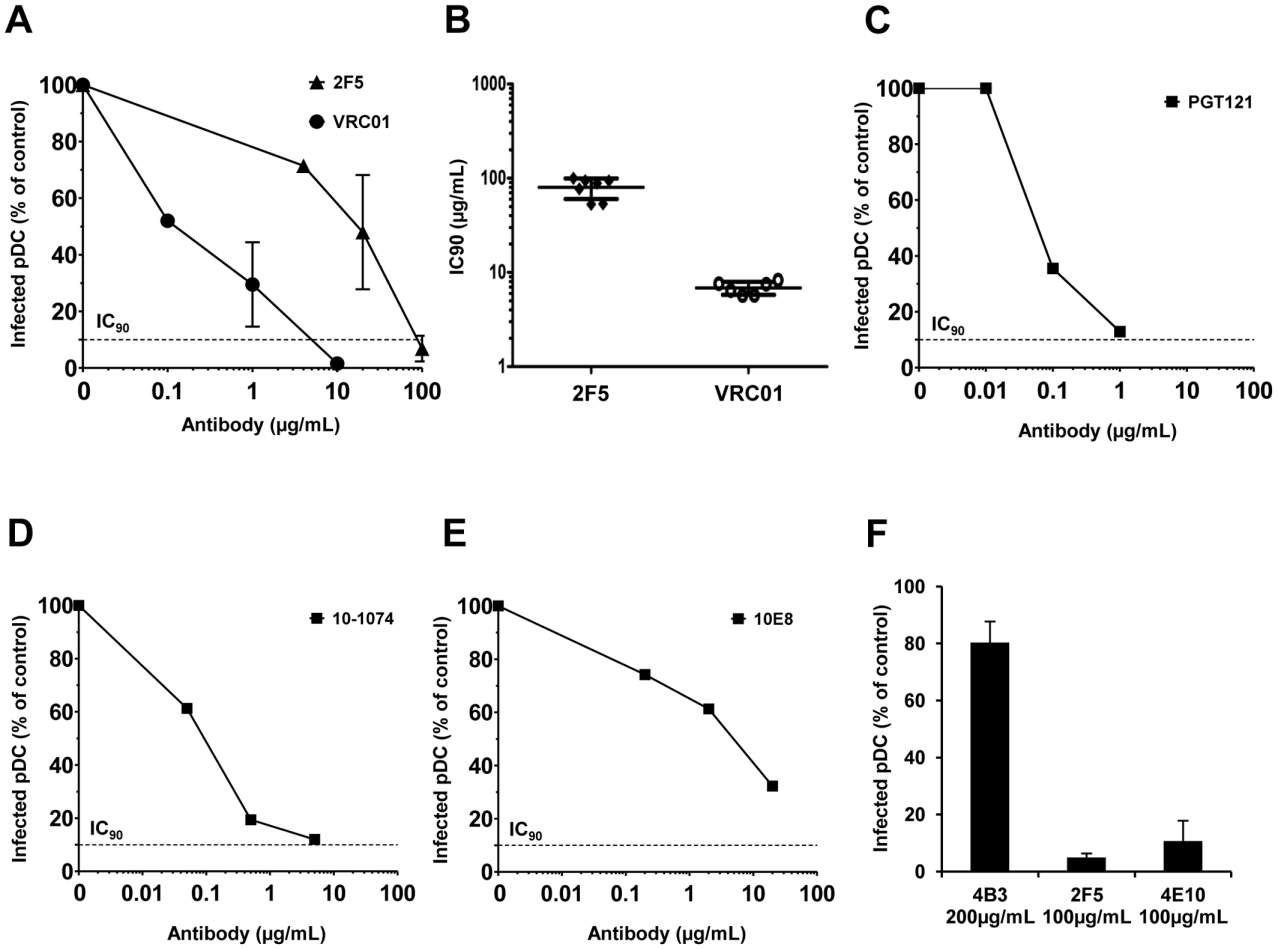
Antibody-mediated inhibition of pDC infection. (A) The percentage of infection of pDC by HIV-1_BaL_ in the presence of various concentrations of NAb VRC01 and 2F5 compared to control without NAb is represented. The IC_90_ (dotted line) corresponds to the concentration of Ab needed to inhibit 90% of the infection. (B) The IC_90_ (µg/ml) measured with NAb 2F5 and VRC01 are represented. Data are the means ± SD of independent experiments performed with cells from 2F5 (n = 7 donors) and VRC01 (n = 6 donors) for (A) and (B). The percentage of infection of pDC by HIV-1_BaL_ in the presence of the most recent broadly anti-gp120 NAb PGT121 (C), 10-1074 (D) and anti-gp41 NAb 10E8 (E) compared to control without Ab are represented. Results of one representative experiment. (F) The percentage of infection of pDC by HIV-1_BaL_ in the presence of anti-gp41 Ab 4B3, 2F5 and 4E10 compared to control without Ab is represented. Ab were tested together on pDC from two different healthy donors and data represent the means ± SD of two independent experiments.

The inhibitory activity of NAb on pDC infection was analyzed. The percentage of HIV-1_BaL_-infected pDC in the presence of NAb compared to infected pDC in the absence of NAb (infection control) was represented for various concentrations of NAb 2F5 and VRC01 (Fig. 2A). The concentration of Ab required to inhibit 90% of the infection (IC_90_) was determined for each anti-HIV-1 Ab and the mean of the IC_90_ from different healthy donors was calculated. The mean IC_90_ of NAb 2F5 and VRC01 were of 80 µg/ml and 7 µg/ml, respectively (Fig. 2B). Moreover, the most recent broadly anti-gp120 NAb PGT121 and 10-1074 efficiently inhibited pDC infection by HIV-1_BaL_ with an IC_90_ of 1.3 µg/ml and 6 µg/ml, respectively; anti-gp41 NAb 10E8 also inhibit pDC infection but with lower efficiency (Fig. 2C, D and E, and Table 1). Moreover, the inhibitory activity of NAb was confirmed on pDC infected with subtype C HIV-1_TV1_ (e.g., IC_90_ of 100 µg/ml for 2F5) and with transmitted/founder HIV-1_Bx11_ viruses^31^ (e.g., IC_90_ of 3.5 µg/ml for PGT121). In parallel, we analyzed the inhibitory activity of these Ab on MoDC infection using similar experimental conditions as for pDC^17^. Interestingly, we observed a 7-80 fold lower IC_90_ on MoDC (Table 1), indicating that pDC infection was more refractory to Ab neutralization than that of MoDC.

We next measured the inhibitory activity of non-neutralizing HIV-1 specific Ab 246-D, 4B3 and 3D6. These Ab recognize the principal immunodominant domain (PID) of gp41. Anti-PID Ab 246-D and 4B3 are NNIAb and inhibited the infection of MoDC with an IC_90_ of 45 µg/ml (Table 1). Despite the inhibitory activity on MoDC, these anti-PID Ab had no effect on pDC infection even at a concentration of 200 µg/ml (Fig. 2F and Table 1). Ab 3D6 did not display any inhibitory activity on the infection of MoDC or pDC by HIV-1, and thus was referred to as a non-neutralizing non-inhibitory Ab (NNNIAb) (Table 1). Altogether, these results showed that infection of pDC by HIV-1 is inhibited by NAb, but not by NNIAb. These results suggest that FcγRIIa is not involved in the inhibition of pDC infection by HIV-1.

### FcγRIIa Does Not Participate in HIV-1 Inhibition by Antibody on Primary pDC

**Figure 3:**
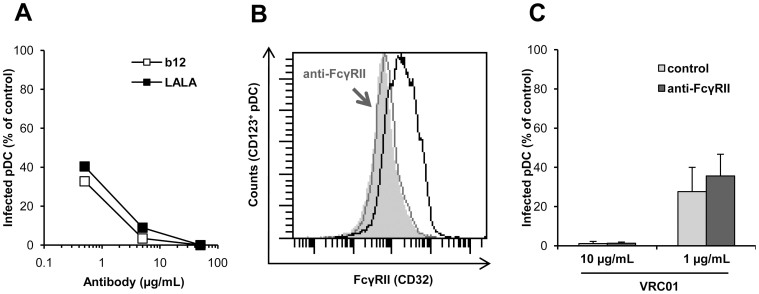
Role of FcγRII in the inhibition of infection of primary pDC by HIV-1. (A) Data represent the percentage of pDC infected by HIV-1_BaL_ in the presence of various concentrations of NAb b12 or LALA (µg/ml) compared to the control condition without NAb. Data are representative of three independent experiments with primary pDC from three different healthy donors. (B) Detection of FcγRII on pDC (black line) or after 30 minutes of incubation with anti-FcγRII Ab (10 µg/ml) (grey line, arrowhead); isotype control in grey. (C) pDC were pre-incubated with or without anti-FcγRII Ab (10 µg/ml) for 30 minutes at 37°C before being added to the HIV-1_BaL_/VRC01 mix. Percentage of infected cells in the presence of NAb VRC01 compared to the control of infected cells in the absence of NAb is shown. Data are means ± SD of two independent experiments performed with primary pDC purified from blood of two healthy donors.

In order to determine the involvement of FcγRIIa in Ab-mediated inhibition, the inhibitory effect NAb b12 and its mutant LALA that is unable to bind to FcγRs^12^ was compared on HIV-1 replication in pDC. We found that LALA and b12 similarly inhibited HIV-1 replication in pDC (Fig. 3A), indicating that HIV-1 inhibition was independent of the Fcγ-FcγR interaction on pDC. In addition, competition experiments were performed with anti-FcγRII Ab on primary pDC. We checked that 10 µg/ml of anti-FcγRII fully blocked further Ab binding (Fig. 3B). pDC pre-incubated with 10 µg/ml of anti-FcγRII were infected with HIV-1_BaL_ in the presence or absence of NAb VRC01. We observed a similar neutralizing activity of VRC01 on HIV-1 replication whether pDC were pre-incubated or not with an anti-FcγRII Ab (Fig. 3C). Altogether, these data demonstrated that FcγRIIa at the surface of pDC was not involved in the inhibition of HIV-1 by NAb.

### Cytokines and Chemokines Production by Primary pDC Is Maintained When HIV-1 Replication Is Inhibited by NAb

**Figure 4:**
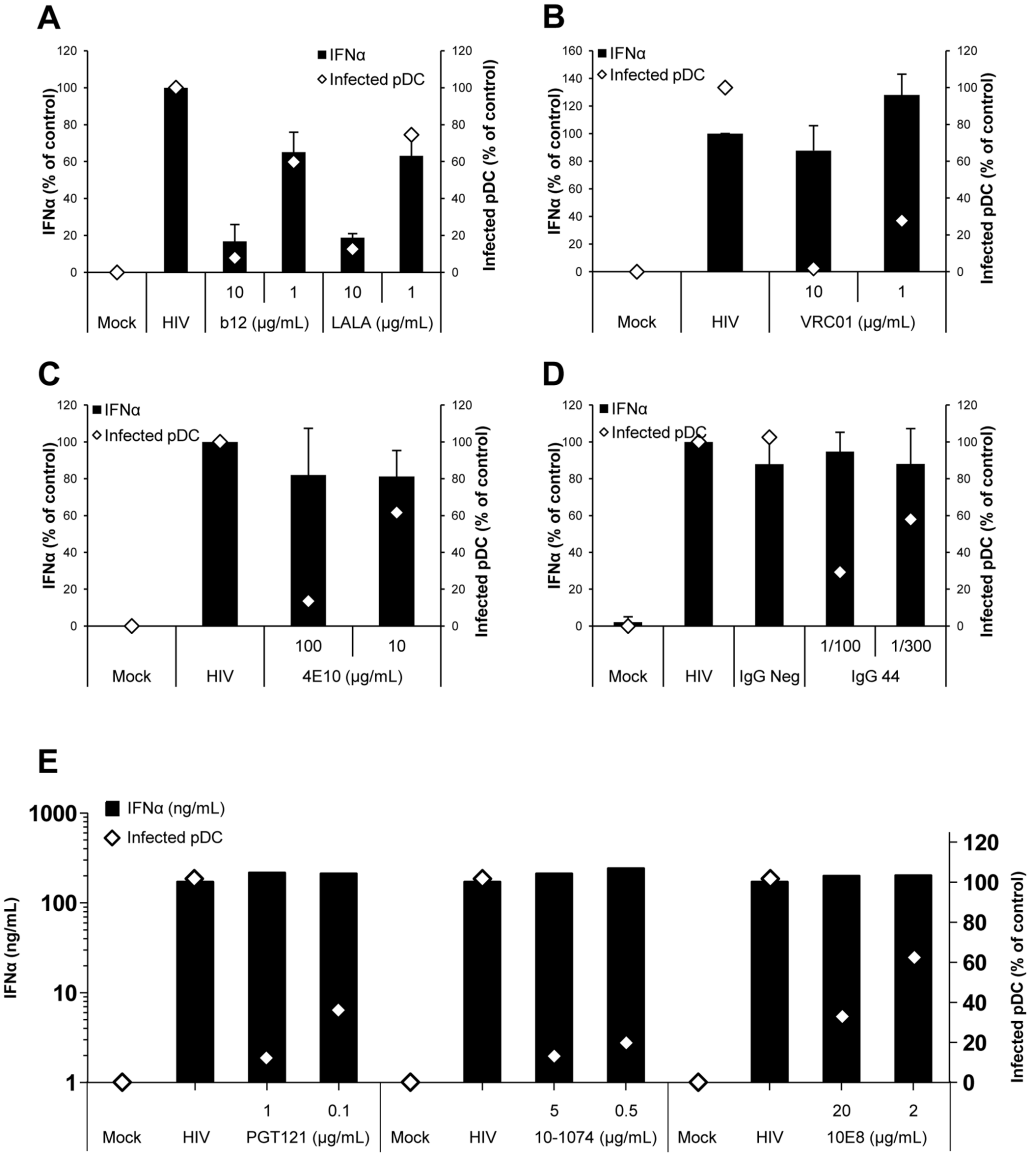
Production of IFN-α by primary pDC during neutralization assay. The supernatants of pDC infected in the presence or absence of different Ab were collected at 48 h post-infection. Histograms represent the concentration of IFN-α detected in the supernatants (black bars, left axis) of pDC incubated alone (Mock) or with HIV-1_BaL_ (HIV) or with HIV-1_BaL_ in the presence of NAb b12 or LALA (A), VRC01 (B), 4E10 (C), IgG purified from serum of HIV-seronegative (IgG Neg) and HIV-1-posive (IgG44) individuals (D), and the most recent broadly NAb PGT121, 10-1074 and 10E8 (E). In parallel, the percentage of infected pDC in the presence of NAb compared to the control condition without NAb is shown (triangle, right axis). Data are means ± SD for IFN-α (ng/ml), representative of three independent experiments performed with primary pDC from three healthy blood donors.

**Figure 5:**
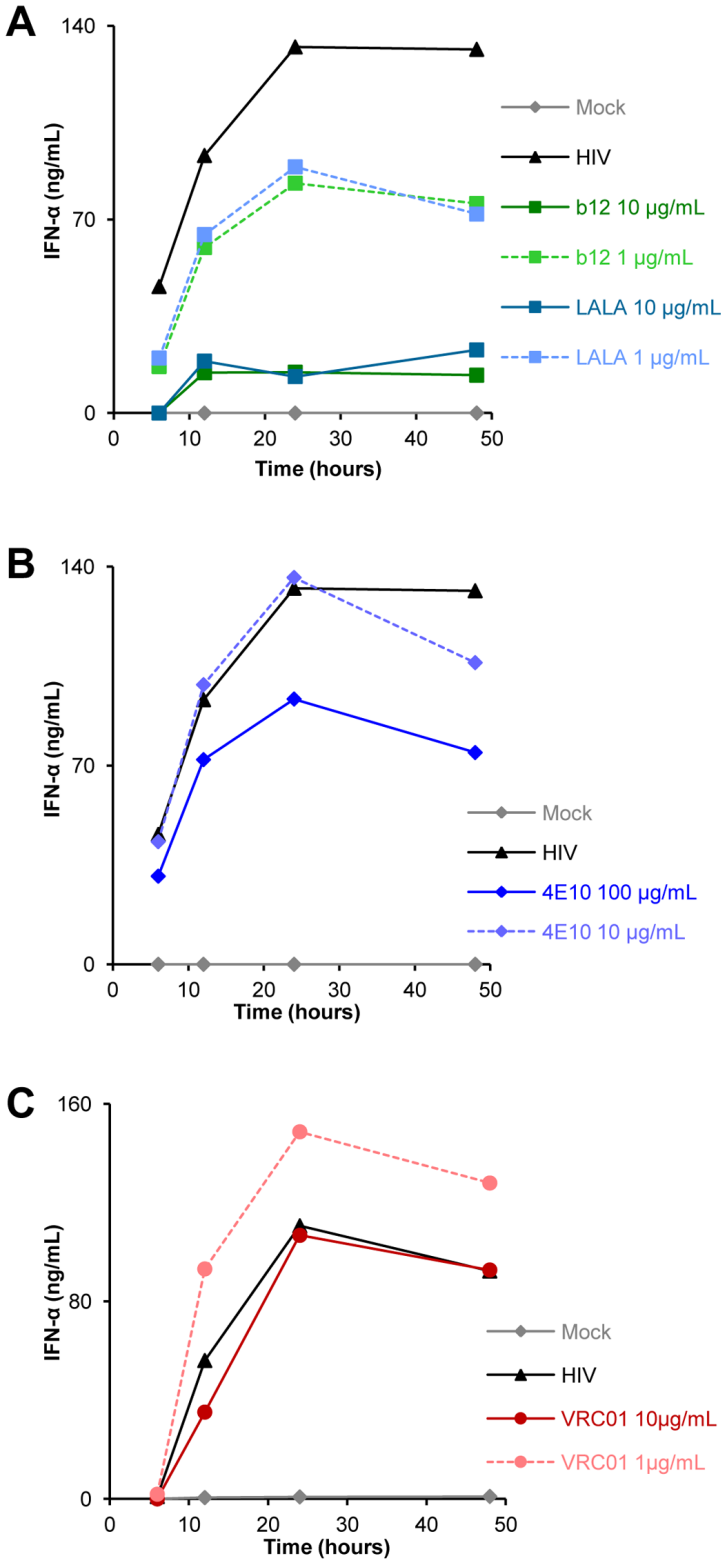
Kinetics of IFN-α production by primary pDC during neutralization assay. Supernatants of the neutralization assays performed with pDC were collected 6, 12, 24 and 48 h and screened by ELISA for IFN-α. The concentration of IFN-α was measured in the supernatants of pDC uninfected (Mock) or incubated with HIV-1_BaL_ (HIV) or incubated with HIV-1_BaL_ in the presence of two concentrations of NAb b12 and LALA (A), 4E10 (B), or VRC01 (C). Data are representative of three independent experiments performed with primary pDC from three healthy blood donors.

The production of IFN-α by primary pDC in the presence or absence of NAb was measured. No or very little IFN-α was produced in the absence of HIV-1 (Mock) or in the presence of NAb alone (no IFN-α detected by ELISA), but following HIV-1_BaL_ infection, IFN-α was detected in the supernatants and this production varied between 20 and 600 ng/ml depending on the donors (Fig. 4). IFN-α production was strongly inhibited in the presence of anti-gp120 NAb b12 at concentrations over 10 µg/ml (Fig. 4A). The mutant version of the b12 Ab, LALA, similarly inhibited IFN-α production (Fig. 4A), indicating that FcγRII was not involved in this inhibition of IFN-α production. Interestingly, NAb VRC01, also directed against the CD4 binding site of the gp120, did not inhibit IFN-α production at viral inhibitory concentrations (Fig. 4B). Similarly, anti-gp41 NAb 4E10 or most recent broadly NAb PGT121, 10-1074 and 10E8 inhibited HIV-1 replication in pDC but did not induced IFN-α production decrease (Fig. 4C and 4E). Polyclonal IgG44 purified from infected patients inhibit HIV-1_BaL_ infection of pDC without modifying the IFN-α production of the cells (Fig. 4D). Moreover, IFN-α secretion (100 ng/mL in control infected pDC) was not impaired following inhibition of transmitted/founder HIV-1_Bx11_ infection by broadly NAb PGT121 and 10-1074 (258 and 253 ng/mL, in PGT121 and 10-1074 treated pDC respectively). Kinetics experiments showed that IFN-α production by pDC started as soon as 6 h post-infection in the presence of HIV-1 and that the inhibition of IFN-α production observed with b12 and LALA at 48 h was the result of an early decrease in the cytokine production (Fig. 5). Therefore, inhibition of IFN-α production was observed following the treatment with the NAb b12 (and LALA) but not systematically observed following inhibition of pDC infection by other NAb. The complete protection of pDC from HIV-1 infection by the NAb was dissociated from the IFN-α production of these cells.

**Figure 6:**
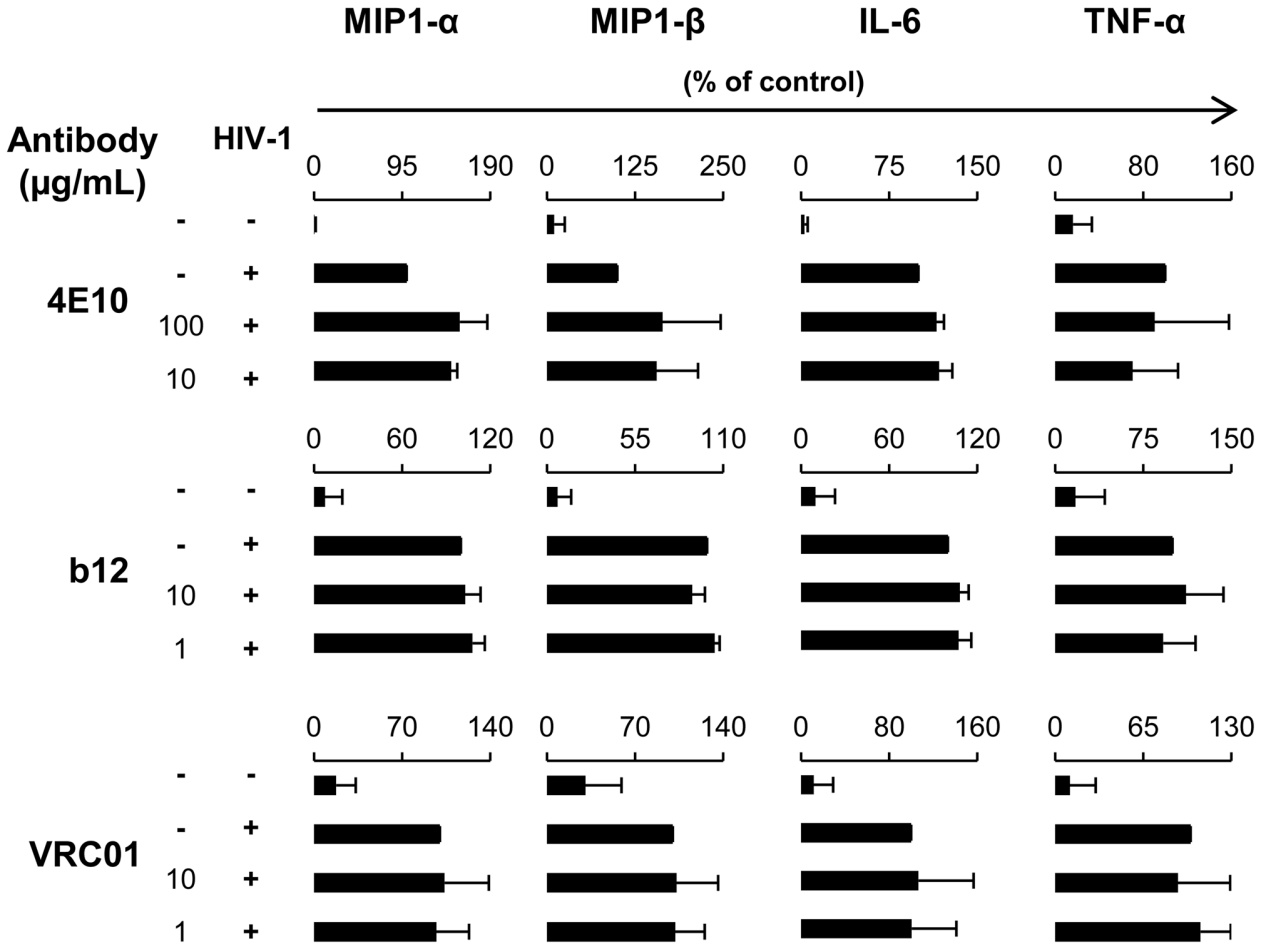
Production of MIP1-α, MIP1-β, IL-6 and TNFα by primary pDC during neutralization assay. Supernatants of pDC were collected after 48 h of infection and screened by CBA Flex to measure MIP1-α, MIP1-β, IL-6 and TNFα. The concentration of MIP1-α, MIP1-β, IL-6 and TNFα in the supernatants from pDC incubated alone (Mock) or with HIV-1_BaL_ (HIV) or with HIV-1_BaL_ in the presence of NAb 4E10, b12 or VRC01 is shown. Data are representative of three independent experiments performed with primary pDC from three healthy blood donors.

**Figure 7:**
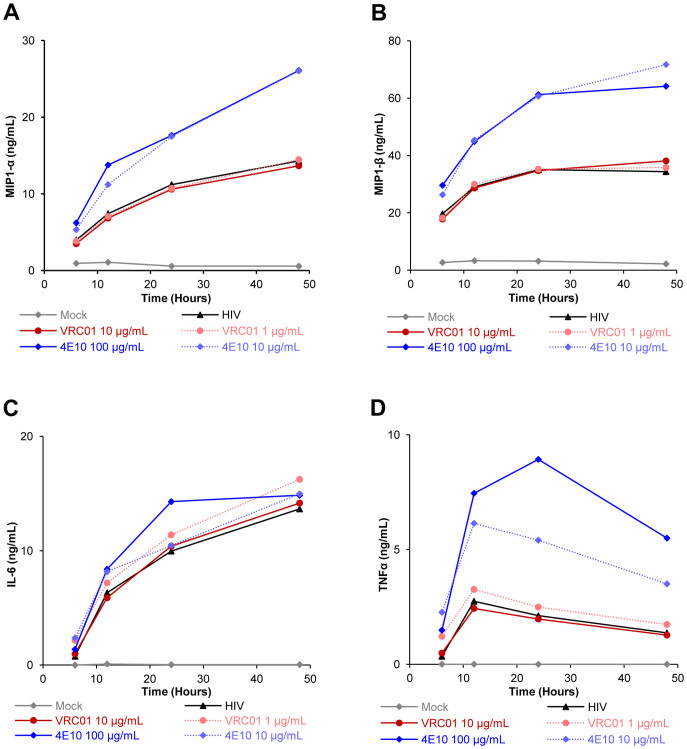
Kinetics of MIP1-α, MIP1-β, IL-6 and TNFα production by primary pDC during neutralization assay. The concentration of MIP1-α (A), MIP1-β (B), IL-6 (C) and TNFα (D) were determined by CBA Flex in the supernatants of pDC collected at different time (6, 12, 24 and 48 h) following incubation without virus (Mock) or with HIV-1_BaL_ (HIV) or with HIV-1_BaL_ in the presence of two concentrations of NAb 4E10 or VRC01. Data are representative of three independent experiments performed with primary pDC from three healthy blood donors.

Besides, we analyzed the presence of other cytokines and chemokines (MIP-1α, MIP-1β, IL-6, and TNF-α) in the supernatants of infected pDC in the presence or absence of NAb. HIV-1 induced the production of these four cytokines/chemokines and the presence of NAb did not decrease this production (Fig. 6). Remarkably, in the presence of viral inhibitory concentrations of NAb 4E10, the production of MIP-1α and MIP-1β was slightly increased (Fig. 6). This increase was not due to the presence of this NAb by its own as 100 µg/ml of 4E10 did not induce the production of MIP-1α or MIP-1β in the absence of HIV-1 (below 5 ng/ml). The increased MIP-1α/β production started as early as 6 h post-infection as observed in kinetics experiments (Fig. 7). The production of IL-6 or TNF-α by pDC was almost unaltered by viral inhibitory concentrations of NAb VRC01, b12 or 4E10. Altogether, these data showed that pDC maintained (or slightly increased) their production of pro-inflammatory cytokines/chemokines MIP-1α, MIP-1β, IL-6, and TNF-α in the presence of these NAb even though they inhibited HIV-1 replication.

## Discussion

In this report we analyzed the capacity of NAb and of some NNIAb to inhibit HIV-1 infection of pDC. We demonstrated that NAb, but not NNIAb, inhibited primary pDC infection by HIV-1. Interestingly, these inhibitory activities were lower than those observed for MoDC infection. We previously showed that NAb inhibitory activity on MoDC was partially attributed to an Fc-mediated inhibitory activity involving the binding of Abs to FcγRII on MoDC^17^. MoDC express both FcγRIIa and FcγRIIb^17^,^18^. The specific expression of FcγRIIa on a subset of primary pDC allowed deciphering the role of the activator FcγRIIa on HIV-1 inhibition. We demonstrated that FcγRIIa was not involved in FcγR-mediated inhibition of HIV-1 replication in primary pDC. First, the NAb b12 and its mutant version LALA, which is unable to bind to FcγRs, inhibited the infection of pDC by HIV-1 with a similar efficacy. Second, blocking the FcγRIIa at the surface of pDC during neutralization assays did not impede the activity of NAb VRC01. Third, NNIAb anti-PID 246-D and 4B3 that inhibit HIV-1 replication in MoDC *via* the participation of FcγRII expressed on these cells, did not inhibit HIV-1 infection of pDC (Table 1). Consistent with our results, induction of the expression of the different FcγRs in the TZM-bl cell line (used for standardized assessment of HIV-1 neutralization) has revealed that FcγRIIb participates in the inhibition of HIV-1 by anti-gp41 Ab whereas the involvement of FcγRIIa was negligible^32^. These results suggest a complex interplay leading to Fc-mediated inhibitory activity. Fc-mediated inhibition of Abs was observed on Langerhans and interstitial DC expressing activator FcγRIIa and inhibitor FcγRIIb^20^. Whether primary mDC display similar Fc-mediated Ab inhibitory activity needs to be further assess. Moreover, it will be valuable to assess whether a balance between activator FcγRIIa and inhibitor FcγRIIb is necessary for such inhibitory activity and whether the lack of Fc-mediated inhibition in pDC was due to an impairment of FcγRs functions in these cells.

pDC actively participate in early innate production of cytokines and chemokines. We analyzed the production of IFN-α, MIP-1α/β, IL-6, and TNF-α in the presence of anti-HIV-1 specific Ab. The production of MIP-1α/β by pDC was increased in the presence of HIV-1_BaL_ infection and NAb 4E10 compared to HIV-1_BaL_ alone. Previously, an increased production of MIP-1α/β has been observed in PBMC treated with anti-cardiolipin Ab^33^. The polyspecific properties of 4E10 may allow this Ab to bind to cardiolipins in addition to HIV-1 gp41 protein^34^, further accounting for this increased production of cytokines.

Remarkably, we found that the induction of IFN-α production by HIV-1 was preserved in the presence of some NAb. Indeed, IFN-α production was detected in the presence of viral inhibitory concentrations of NAb VRC01, 4E10, PGT121, 10-1074, 10E8 and polyclonal IgG44. These results contrast with the recent reports showing a decreased IFN-α production in the presence of viral inhibitory concentrations of VRC01 and 10-1074 using other experimental conditions, ie PBMC as source of pDC co-cultured with HIV-1-infected MT4C5 lymphoblastoid T cells and detection of type-I IFN with reporter cell line HL116^35^. However, we observed an inhibition of IFN-α production by NAb b12 (and its mutant LALA), consistent with previous reports using the same NAb^26^. Indeed, it was suggested that the inhibition of IFN-α production was the consequence of interaction between viral gp120 and cellular CD4^26^,^36^,^37^. Interestingly, in our study, NAb VRC01 that recognizes the CD4 binding site (CD4bs) of the gp120^38^ and polyclonal IgG (that probably contains a large number of anti-CD4bs Ab)^39^, had almost no effect on IFN-α production by primary pDC while they inhibited the productive infection of the cells. As these Ab interact with the CD4bs of gp120, we may hypothesize that interaction leading to IFN-α production is not hindered by NAb VRC01 on primary pDC. Indeed, NAb VRC01 binds to a CD4bs epitope distinct to that of b12 and do not induce the shedding of gp120 like b12^40^. In addition, large amounts of noninfectious HIV-1 may contribute to IFN induction by pDC through induction of autophagy following TLR7 signaling^41^. These noninfectious viruses may be differentially inhibited by NAb depending to the epitope recognized. Inversely, Fc portion of IgG could downregulate virus-induced IFN-α production by pDC^42^. Therefore, mechanisms leading to IFN-α inhibition will depend on the nature of the stimuli induced both by cell surface receptors and by intracellular pathways^43^. IFN-α production in the presence of virus/IgG immune complexes has been previously reported with other viruses^44^,^45^,^46^. For HIV-1 infection, additional studies will be necessary to define the specific Ab feature and the mechanism by which they will selectively inhibit or maintain IFN-α production by pDC. As IFN-α displays antiviral function, it may participate in HIV-1 inhibitory activity. In this study, the protective benefit of IFN-α released in the supernatant could not been assessed as primary pDC can be maintained only in culture for a few days. However, *in vivo*, pro-inflammatory cytokines released by pDC may contribute to HIV-1 elimination in addition to direct inhibition of HIV-1 replication by NAb. Whether IFN-α production by pDC during the chronic phase is detrimental or may contribute to pathogenesis is still a matter of debate^47^,^48^,^49^,^50^. Recent studies however suggest that IFN-α release during the acute phase may not play a direct pathologic role in HIV-1 disease progression^47^,^48^.

We showed here that the polyclonal IgG44, as well as anti-HIV-1 NAb VRC01, 4E10, PGT121, 10-1074, 10E8, but not b12, failed to decrease the IFN-α production induced by HIV-1 at viral inhibitory concentrations. These findings suggest that, in addition to direct inhibition of HIV-1 replication by NAb, the maintenance of pro-inflammatory cytokines and chemokines released by pDC may contribute to HIV-1 elimination by triggering the innate immune responses and thus impeding early HIV-1 dissemination. Therefore, in the context of a prophylactic vaccine development, induction of anti-HIV-1 NAb that simultaneously protect HIV-1 target cells and allow innate immune cells to maintain an antiviral immune response, including IFN-α production, should be favored.

## Methods

### Primary DC Preparation

Briefly, pDC were isolated from human PBMC collected from healthy anonymized HIV-1-seronegative blood donors (EFS, Strasbourg, France) by magnetic purification using an AutoMACS and BDCA-4 MicroBeads kits (Miltenyi Biotec, Cologne, Germany). The FcγRIIa^+^ population was recovered following BDCA-4 positive selection, as previously reported^51^. pDC were then cultured overnight at 10^6^ cells/ml in RPMI 1640 + GlutaMAX^TM^ (GIBCO) supplemented with 10% fetal calf serum (FCS) and 1 ng/ml of interleukin-3 (IL-3) (R&D Systems, Minneapolis, USA) before any experiment at 37°C, under 5% CO_2_. For MoDC preparation, similarly as pDC, monocytes were purified from PBMC by magnetic purification using CD14 MicroBeads kit (Miltenyi Biotec), as previously described^17^,^21^.

### Virus Preparation

Virus stocks for each primary isolate were obtained by amplification on PBMC from seronegative donors, as previously described^17^,^21^. The HIV-1_BaL_ (subtype B, R5 strain) was provided by Drs. S. Gartner, M. Popovic, and R. Gallo from the National Institutes of Health (NIH). This virus was only amplified on PBMC and therefore considered as a primary isolate. HIV-1_TV1_ (subtype C, R5 strain) primary isolate was obtained from Dr. S. Engelbrecht (NHLS, Tygerberg, South Africa). Transmitted/Founder HIV-1_Bx11_ primary isolate was obtained before seroconversion from French HIV-infected individual as previously described^31^.

### HIV-1 Specific Antibodies

Monoclonal anti-HIV-1 human Ab to gp120 IgG1 b12, LALA and PGT121 were kindly provided by Dr. D. Burton and Dr. P. Poignard (The Scripps Research Institute, La Jolla, CA). Monoclonal anti-HIV-1 human Ab to gp120 IgG1 10-1074 and gp41 10E8 were kindly provided by Dr. H. Mouquet (Institut Pasteur, Paris). Human monoclonal Ab 447-52D (anti-gp120), and 246-D (anti-gp41) were kindly provided by Dr. S. Zolla-Pazner (New York University School of Medicine, NY). Human monoclonal Ab 4E10, 2F5, 4B3, 3D6 (anti-gp41), and 2G12 (anti-gp120) were kindly provided by Dr. D. Katinger (Polymun Scientific GmbH, Vienna, Austria). Human monoclonal Ab VRC01 (anti-gp120) was kindly provided by Dr. J. R. Mascola (NIH, Bethesda, MD). Human polyclonal IgG were purified from sera of asymptomatic HIV-1 patients (approval obtained from the Comité Consultatif pour la Protection des Personnes dans la Recherche Biomédicale [CCPPRB], and informed consent was provided according to the declaration of Helsinki)^52^. And the methods were carried out in accordance with the approved guidelines.

### HIV-1 Inhibitory Assays

Aliquots of 25 µl of HIV primary isolate were incubated with 25 µl of Ab for 1 hour (h) then 25 µl of either pDC (10 × 10^6^ cells/ml) or MoDC (15 × 10^6^ cells/ml) was added. As pDC do not support long-lasting *in vitro* cell culture, a single cycle infection protocol with high titer inoculums was chosen to assess virus replication in these cells. Therefore, HIV-1 primary isolates were used at 1.25 µg/ml and 2 µg/ml of p24 antigen with pDC and MoDC respectively, in order to detect about 3% infected cells. When indicated, HIV-1 reverse transcriptase inhibitor Azidothymidine (AZT, Sigma-Aldrich) was added at 5 µM to the cell culture to inhibit viral replication, allowing the residual p24 to be measured in the absence of *de novo* viral synthesis. After 48h, various cell surface markers and intracellular HIV-1 p24 antigen protein were stained to characterize the cells (pDC as CD123^+^, and MoDC as CD209^+^) and to determine the percentage of infected cells by flow cytometry. The concentration of Ab required to inhibit 90% of cell infection (IC_90_) was determined^17^. To analyze the role of FcγRs in HIV-1 inhibition, pDC were incubated with 10 µg/ml of purified anti-FcγRII Ab (BD Pharmingen) for 30 minutes prior to addition to the HIV-1/Ab immune complexes.

### Immunophenotyping and Flow Cytometry Analysis

Briefly, cells were labeled with the Live/Dead Fixable Dead Cell Stain kit (Invitrogen/Applied Biosystems, CA) for 10 minutes at room temperature. Mouse monoclonal fluorescent Ab against human CD123-Pe-Cy5 (BD Pharmingen, USA), anti-human CD32-FITC (BD Pharmingen), anti-human CD209-PerCP-Cy5.5 (BD Pharmingen) and anti-human CD83-APC (Miltenyi Biotec) were added and the samples incubated for 10 minutes at 4°C. Cells were fixed and permeabilized using the Cytofix and Perm/Wash solutions (BD Biosciences). Then, intracellular p24 antigen was stained using anti-HIV-1 core protein p24 RD-1 (Beckman Coulter). Multi-color samples were acquired on a LSRII SORP cytometer (BD Biosciences) calibrated using Cytometer Setup & Tracking beads (BD Biosciences) to ensure consistency of fluorescence intensity measurement throughout all experiments. Compensation was performed with a CompBeads kit (BD Biosciences). Doublet cells were excluded using Forward Scatter Width and Forward Scatter Area and dead cells were excluded using Live/Dead staining. FACSDiva™ software version 6.1.2 (BD Biosciences) was used for the final analysis and graphical output.

### Cytokines and Chemokines Analysis

Supernatants were collected at various time points during inhibitory assays for analysis of cytokines and chemokines production. IFN-α was measured using Verikine Human Interferon Alpha MultiSubtype ELISA kit (PBL InterferonSource, NJ). MIP-1α, MIP-1β, IL-6 and TNF-α were measured with Cytometric Bead Array (CBA, BD Biosciences) using appropriate Flex sets and Human Soluble Protein Master Buffer kit (BD Biosciences), acquired on a LSRII SORP cytometer, and analyzed using FCAP Array software version 1.0.1 (Soft Flow, Hungary).

### Antibody Binding Assay

pDC were incubated with or without 10 µg/ml of purified anti-FcγRII (CD32) Ab (BD Biosciences) for 30 minutes at 37°C, then labeled with anti-CD32 FITC (BD Biosciences) for 10 minutes at 4°C. The samples were fixed in Cytofix solution, acquired by LSRII SORP cytometer and analyzed using FACSDiva™ software version 6.1.2.

### Statistical Analysis

Groups were compared by one-way ANOVA (Kruskal-Wallis test). Values of *P* < 0.05 were considered to be statistically significant. All statistical calculations were performed using GraphPad Prism 5.04 software (GraphPad, CA).

## Author Contributions

A.L. and B.S. performed the experiments; A.L., B.S., V.H. and C.M. analyzed the data, A.L., B.S., V.H., J.P., S.S., T.D., G.L. and C.M. contributed to reagents/materials/analysis tools; A.L., B.S., V.H. and C.M. conceived, designed the experiments; A.L., B.S. and C.M. wrote the paper. All authors approved the final manuscript.

## Figures and Tables

**Table 1:** Inhibitory activity of NAb and NNIAb when primary pDC/MoDC were used as HIV-1_BaL_ target cells. IC_90_: concentration (µg/ml) of Ab necessary to reduced by 90% the number of infected cells; -, no inhibitory activity detected at 100 µg/ml of Ab. NNNIAb, non-neutralizing non-inhibitory anti-HIV-1 Ab. Values are the mean of 3–7 different independent experiments performed on cells purified from different independent healthy blood donors

	IC_90_ (µg/ml)								
	**NAb**								
	2F5	4E10	10E8	b12	2G12	447-52D	VRC01	PGT121	10-1074
pDC	80	100	100	8	10	80	7	1.3	6
MoDC	1	5	20	1	1	2	1	0.2	0.6
	**NNIAb**		**NNNIAb**						
	246-D	4B3	3D6						
pDC	-	-	-						
MoDC	45	45	-						
